# Primary health Centres’ performance assessment measures in developing countries: review of the empirical literature

**DOI:** 10.1186/s12913-018-3423-0

**Published:** 2018-08-09

**Authors:** R. Bangalore Sathyananda, A. de Rijk, U. Manjunath, A. Krumeich, C. P. van Schayck

**Affiliations:** 1 Department of Primary Care, CAPHRI, Maastricht University, Maastricht, The Netherlands; 2 Department of Social Medicine, research institute CAPHRI, Maastricht University, Maastricht, The Netherlands; 3 Institute of Health Management Research, Bangalore, India; 4 Department of Health Ethics and Society, research institute CAPHRI, Maastricht University, Maastricht, The Netherlands; 5 Present address: No 18, 3rd Main, 1stCross, Navodaya Layout, Shakambari Nagar, Sarakki, J P Nagar 1st Phase, Bengaluru, Karnataka 560070 India

**Keywords:** Primary health centres, Performance assessment, Developing countries

## Abstract

**Background:**

It is universally accepted that primary healthcare is essential for achieving public health and that assessment of its performance is critical for continuous improvement. The World Health Organization’s (WHO’s) framework for performance assessment is a comprehensive global standard, but difficult to apply in developing countries because of financial and data constraints. This study aims to review the empirical literature on measures for Primary Health Centre (PHC) performance assessment in developing countries, and compare them for comprehensiveness with the aspects described by the WHO Framework.

**Methods:**

Research articles published in English scientific journals between January 1979 and October 2016 were reviewed systematically. The reporting quality of the article and the quality of the measures were assessed with instruments adapted for the purpose of this study. Data was categorized and described.

**Results:**

Fifteen articles were included in the study out of 4359 articles reviewed. Nine articles used quantitative methods, one article used qualitative methods exclusively and five used mixed methods. Fourteen articles had a good description of the measurement properties. None of the articles presented validity tests of the measures but eleven articles presented measures that were well established. Mostly studies included components of personnel competencies (skilled/ non-skilled) and centre performance (patient satisfaction/cost /efficiency).

**Conclusions:**

In comparison to the WHO framework, the measures in the articles were limited in scope as they did not represent all service components of PHCs. Hence, PHC performance assessment should include system components along with relevant measures of personnel performance beyond knowledge of protocols. Existing measures for PHC performance assessment in developing countries need to be validated and concise measures for neglected aspects need to be developed.

**Electronic supplementary material:**

The online version of this article (10.1186/s12913-018-3423-0) contains supplementary material, which is available to authorized users.

## Background

During the last three decades, significant achievements have been made in improving the health of the world population [1, 2]. This can be attributed in part to the Millennium Development Goals [1] and is further augmented by the launch of the Sustainable Development Goals in September 2015 [2]. In developing countries, however, still more progress needs to be made. In these countries, still far too many women die during childbirth [1–5], too many children die from preventable causes [1, 2, 4, 6] and too many adults die from treatable infectious and non-communicable diseases [6, 7]. Reducing mortality and morbidity is the main focus of primary healthcare [1, 2]. While progress has been made in communicable diseases, the burden of non-communicable diseases is straining developing countries’ healthcare resources [6, 7].

Primary healthcare is an essential and critical type of healthcare delivery that addresses the health needs of the population usually delivered at centres called Primary Health Centres (PHC) [8]. In the last decade, the achievements of healthcare have gained significant attention as the ‘performance of healthcare’ which is not specific to PHC [9–14]. In order to monitor their health system, countries carry out performance assessment; the performance is fulfilling one’s obligation, in a way that releases one from liabilities [15]. Performance assessment can be defined as a ‘coherent evaluation system which assesses the whole occupational functioning including its constituent parts’ [16]. A comprehensive assessment of the system in developing countries is vital for determining the gap between demand for services and the ability of the healthcare systems to reciprocate.

In this regard, the World Health Organization’s (WHO) health systems performance assessment framework serves as the global benchmark. According to the framework, health systems performance objectives are good health, responsiveness and fair financial contribution [17]. The framework describes six aspects of performance assessment: *overall level of health* considering the general health of population; *distribution of health in population* (healthcare services coverage); *overall level of responsiveness* indicated by the quality of care, satisfaction of care and availability of services; *distribution of responsiveness*; *distribution of resources* such as human resources and care facilities; *distribution of financial contribution* from various agents and their optimal use [17]. Even though it would be ideal, many developing countries might lack adequate resources and data to assess PHC performance according to this framework, and hence, may have reservations about applying the framework [10, 18].

The specific measures of PHC performance assessment which are used in developing countries are not well known. In the most recent review of health care performance measurements, no distinction was made between developed and developing countries [19]. The Primary Healthcare Systems are evolving at a faster pace in developing countries than before and lacks clarity, hence the Primary Health Care Performance Initiative calls the system’s performance a “Black Box” and identifies an urgent need to build on the existing knowledge [20].

The aim of this study is to review the scientific literature on measures of PHC performance assessment used in developing countries and to compare them with the WHO framework for health care performance assessment for comprehensiveness. Based on the literature, four aspects of healthcare performance measurement [9, 11, 21] are emphasized in this review:

1. The methods for assessing performance in health care [22]
2. The quality of measurement (validity and reliability) [16]
3. The professional actions, that is, the performance of the provider [23–25]
4. The levels covered by the measurement: the level of the patient, that of the community, district/state level and/or country level. Concurrently, measures could capture different perspectives such as that of the health care provider or that of the patient [26].

## Methods

**Fig. 1:**
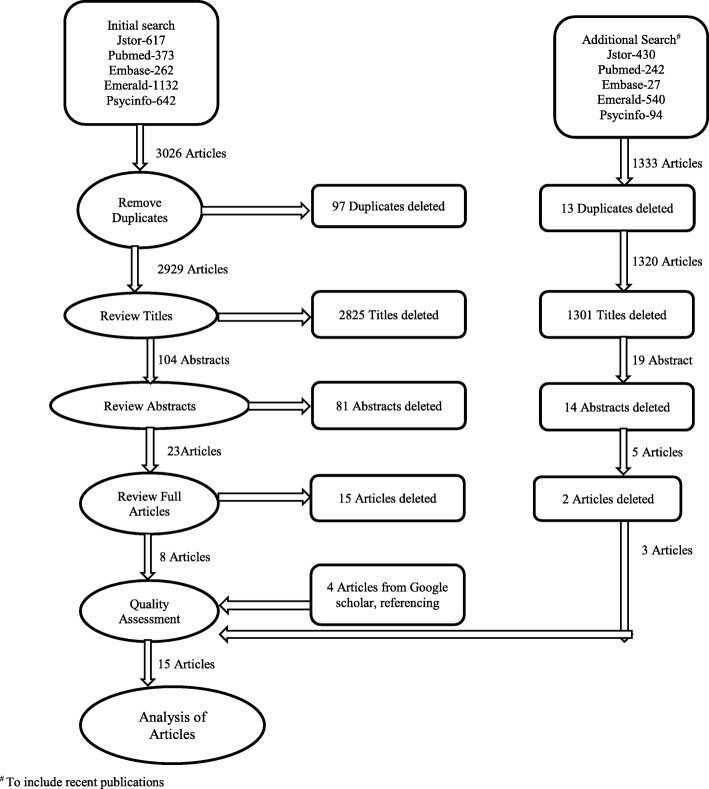
Methodology of Review

This paper employs a review of the literature on PHC performance assessment in developing countries. The flow diagram of the selection and search process is depicted in Fig. 1.

### Search strategy

An initial search for articles from the following databases was carried out: Hospital/Health care (Pubmed, Embase), Psychology (JSTOR, PsycINFO) and Business (Emerald Insight). Endnote7 software was utilised to download and select articles. As the aim of the review was to include articles on PHC performance assessment in developing countries that are published in English, the search terms included ‘primary health centre’ or ‘health centre’ and ‘performance assessment’ or ‘performance’ and ‘developing country’ {(primary health centre or health centre) and (performance assessment or performance) and developing country}. The period of publication for the search was from the year 1979, a year after Alma Ata up to December 2014. Further, an additional search was done from January 2015 to October 2016 to include recent articles. The search terms were tested and Mesh terms were employed during search when available in the database. The search results from the databases identified were combined to eliminate duplicates. The details of the search strategy are given in Additional file 1: Appendices 1 and 2. A similar search was conducted in Google scholar and the articles fulfilling inclusion criteria were included for full review (Fig. 1).

The inclusion criteria employed were:

- Empirical articles with measurement of PHC or professional performance (from professional or health centre perspectives);
- The measurements were conducted in a developing country; and
- Papers were published in English

Articles that reported only on methodology of research, discussion on performance/performance assessment and review of literature were excluded. The authenticity of this review was ensured by including only published empirical articles, avoiding grey literature and reviews. On the basis of these inclusion and exclusion criteria, the articles were screened first by title, then by the abstract and full article, to select the relevant articles for full paper review.

### Quality assessment

Next, the selected full articles were assessed for quality. The complete evaluation of methodological characteristics as suggested by COSMIN and Noben and colleagues could not be applied, as methodologies of the articles selected were not advanced enough [27, 28]. Hence, a simpler instrument was developed, based on the quality of the description and the quality of the measurement property (the reliability and validity) itself. It is theoretically possible that the measurement property is weak although the description is good [28]. With our instrument for assessing the quality of description, the articles were scored as:0, if there was no description of the measurement property;1, if only a few points of the measurement property were described (little information); and 2, if there was a detailed description of the measure in the article (good information).

The quality of the measurement property was assessed based on four criteria:

1. The standardization: method of data collection was noted and the standardization by pretesting of the tool and/or the training of researchers was reported.
2. The triangulation of the data collection method and/or data source with significantly similar results.
3. The measure used was well established/widely published, this was done by verifying references and other cited publications.
4. The reliability and validity of the measures was tested using statistical methods and compared with a global standard (WHO performance assessment framework) [29].

The quality was scored as follows:

- 0 if none of the above criteria was fulfilled
- 1 if only standardization of the measure and/or researchers by training was done
- 2 if triangulation of methods/data sources was done
- 3 if the methods used for analysis were well established/published
- 4 if validity of the measure was tested with good results

The results were relegated to a lower number if the higher criteria were not fully met or exclusively mentioned in the article. The qualitative article was scored between 0 and 2 as its measures would be unique, based on the theoretical framework used (scores 3 and 4 were irrelevant). The papers were analysed for quality by the first two authors independently, the differences were discussed and agreed upon.

### Descriptive analysis

The selected full articles were analysed and reviewed to understand and describe how the performance of PHCs had been assessed in developing countries. All selected articles were studied to understand how they had defined performance and the perspectives of the performance assessment (provider or patient). The topographical and content analysis of measures was done and these were compared with the measures of WHO performance assessment framework for comprehensiveness [29].

### Ethics

No ethical approval was needed for this narrative review.

## Results

### Literature search results

After downloading the articles (4359 articles) using the search strategy described above, duplicates were removed, inclusion and exclusion criteria were applied systematically, which resulted in the selection of eight articles from the initial search and three articles from the additional search for the review. Further, searches from the reference tracking and in Google Scholar resulted in another four articles (Fig. 1). Thus, in total fifteen articles were included for quality assessment and content analysis (Table 1). Table 1 provides an overview of these fifteen articles.

**Table 1:** Description of articles

Sl No	Title	Authors	Publication	Country	Study type	Study population	Size of Population	Definition of Performance	Measures identified	Performance distinguished as centre(system)/personnel	Degree of description of measurement property	Quality of measurement property
**1**	Investing in Improved Performance of National Tuberculosis Programs Reduces the Tuberculosis Burden: Analysis of 22 High-Burden Countries	Akachi, Y., A. Zumla, and R. Atun [28]	The Journal of Infectious Diseases, 2012. 205: p. S284-S292	High TB burden countries	Quantitative	Developing Countries	Not Applicable	1. Secondary analysis of WHO, OECD data 2. Indicators of National Tubercular Program (NTP) and its effect on burden of disease	1. Tuberculosis Burden: a. Incidence b. Prevalence c. Mortality 2. Tuberculosis control program: a. Case detection rate b. Treatment success rate c. NTP expenditure	System (Country)	2	3
**2**	Performance of female volunteer community health workers in Dhaka urban slums	Alam, K., S. Tasneem, and E. Oliveras [29]	Social Science & Medicine, 2012. 75(3): p. 511–515.	Dhaka, Bangladesh	Mixed Method [Quantitative and Qualitative (Focus group discussion)]	Community Health Workers (CHW)	542 (50% of CHW) + 3	Active participation	1. Activities, tasks and services: a. Home visits b. Identifying pregnancies c. Bringing pregnant women to delivery centres d. Accompanying pregnant women to delivery and providing essential new born care	Personnel	2	1
**3**	Accessibility to tuberculosis treatment: assessment of health service performance.	Arakawa, T., et al. [30]	Rev Lat Am Enfermagem, 2011. 19(4): p. 994–1002	Ribeirao Preto, Sao Paulo State, Brazil	Quantitative	Persons with TB and undergoing treatment at referral services	100	Accessibility of services	1. Organization accessibility 2. Economic accessibility 3. Geographical accessibility	Centre	2	3
**4**	Problems measuring community health status at a local level: Papua New Guinea’s health information system.	Ashwell, H.E. and L. Barclay [31]	Rural Remote Health, 2010. 10(4): p. 1539.	Papua New Guinea’	Mixed Method[Qualitative (interviews) Quantitative data from census]	Health persons rendering services at national, provincial and district health facilities	175 + 77	1. Community Health and Wellbeing 2. Community use of services	1. Community Health a. Physical health b. Social and Economic well being c. Healthy lifestyle d. Hygienic living environment e. Maternal and Child Health 2. Use of services a. Use of Antenatal, childbirth, immunization services b. Use of Environmentally induced diseases like malaria, pneumonia diarrhoea	Centre	1	2
**5**	Evaluation of maternal and child health services in El-Minia City, Egypt.	Awadalla, H.I., et al. [32]	Journal of Public Health, 2009. 17(5): p. 321–329.	El-Minia City, Egypt	Quantitative	Female clients using health services at maternal and child health centres	400	1. Utilization 2. Client Satisfaction	1.Utilization of various components of Maternal &Child Health (MCH) services a. Abortion b. Under 5 year mortality c. Curative MCH services d. Antenatal care e. Delivery services f. Family planning services g. Preventive and curative MCH services 2.Satisfaction a. Waiting time b. Environment c. Doctor client interaction d. Nurse client interaction e. Economic feasibility	Centre	2	1
**6**	District health managers’ perceptions of supervision in Malawi and Tanzania.	Bradley, S., et al. [33]	Hum Resour Health, 2013. 11: p. 43.	Malawi and Tanzania	Qualitative	District health management team	57	1. Health indicators 2. Facility Provision 3. Individual Staff performance 4. Supervisory practices	1.Health indicators a. Number of Deliveries b. Maternal mortality Figs. 2. Facility Provision a. Availability of supplies b. Registers filled c. Cleanliness of wards 3. Individual Staff performance a. Punctuality b. Response time for on call staff c. Absenteeism d. Staff reporting to work at recommended time 4. Supervisory practices	Personnel	2	2*
**7**	A Rapid assessment methodology for the evaluation of primary care organization and performance in Brazil	Macinko, J., C. Almeida, and P.K. de Sá [34]	Health Policy and Planning, 2007. 22(3): p. 167–177.	Brazil	Quantitative	Client and provider	936	Assessment of primary care experiences	1.Accessibility of Facility and Services 2.Gate keeping/ First contact care 3.Longitudinality 4.Comprehensiveness 5.Coordination 6.Family focus 7.Community orientation 8.Provider characteristics	Centre	2	3
**8**	The establishment of bonds between professional and patient in TB treatment: the performance of primary health care services in a city in the interior of Sao Paulo	Ponce, M.A., et al. [35]	Rev Lat Am Enfermagem, 2011. 19(5): p. 1222–9	Sao Paul, Brazil	Quantitative	Patient Health professional Managers	108 + 37 + 15	Establishment of bonds (Patient experience Health professional experience Managers experience)	Bonding Identified by 11 items	Centre	2	3
**9**	Assessing the performance of primary health centres under decentralized government in Kerala, India	Varathrajan D, Thankappan R, Jayapalan S [36]	Health Policy and Planning, 2004.19(1)41–51	Kerala, India	Mixed Method [Qualitative (key informant/ client interviews) Quantitative data from PHCs]	Primary Health Centre	10	Cost effectiveness	1. Infrastructure: Building structure, Toilet, Clean running water, Electricity, Communication, Wash basin, equipment and instruments, furniture, drugs and other supplies 2. Access: Size of building to patient load, home visits by PHC staff, facility hours, patient records waiting area, patient privacy, distribution/display of health education materials, display of community statistics 3. Costs: salary, investment, maintenance, patient care, building, furniture, equipment 4. Number of patient contacts served 5. Client experience: focus on illness, service received, access frequency, staff behaviour, diagnosis 6. Key informant experience: budget, cost, financial sources, PHC and local government characteristics and linkages	Centre	2	3
**10**	Gap analysis and the performance of primary health centres in the implementation of the school health programme of NRHM	Shreedevi D [37]	International journal of Research in Business Management, 2014.2(2)1–8	Andhra Pradesh, India	Quantitative	Primary Health Centre	159	Program delivery	Program Specific 1. Services a. Screening, Health care and Referral b. Immunization c. Micronutrient management d. De-worming 2. Promotion 3. Capacity building 4. Monitoring & Evaluation 5. Midday Meal	System(District)	2	3
**11**	Factors affecting the performance of maternal health care providers in Armenia	Fort AL, Voltero L [38]	Human Resources for Health 2004, p 2–8	Armenia	Mixed Method [Qualitative (personnel interviews) Quantitative data (skill items)]	Nurses and Midwifes	285	Completion of clinical and non-clinical tasks	Skill Items of 1. Prenatal care (42 items) 2. Post-natal care (3 items)	Personnel	2	3
**12**	Improving health worker performance: The patient-perspective from a PBF program in Rwanda	Lannes. L [39]	Social science and Medicine (2015). 138:1–11	Rwanda	Quantitative	Health workers of Primary level facilities	157	Patient satisfaction	1. Clinical services a. Privacy during examination b. Staff attitude c. Explanation d. Cost of drugs e. Cost of services f. Availability of drugs g. Overall satisfaction 2. Non-clinical services a. Waiting time b. Time with provider c. Cleanliness	Personnel	2	3
**13**	Assessment of the role of primary health care in tuberculosis control in Serbia	Stosic M, Lazarevic N, Kuruc V, Ristic L [40]	MedicinskiPregled (Novi Sad) 2015. 68(9–10):331–335	Serbia	Quantitative	Primary Health Centre	19	Organization of care	1. Availability and coverage of general practice and TB services 2. Health activities performed 3. Collaboration with health services Compliance to health needs	Centre	2	3
**14**	Skilled Birth Attendants in Tanzania: A descriptive study of cadres and emergency obstetric care signal functions performed	Uneo E, Adegoke A. A, Masenga G, Fimbo J, Msuya S E [41]	Maternal and child health journal, 2015.19:155–169	Tanzania	Mixed Method[Quantitative(facility survey and task items) Qualitative(challenges in care delivery)]	Healthcare workers in Primary Health Centre	158	Knowledge and Skill of Emergency Obstetric Care signal functions	1. BEmOC signal functions a. Administers parenteral antibiotics b. Administers uterotonics drug c. Administers parenteral anti-convulsants d. Manually remove the placenta e. Remove retained products f. Perform assisted vaginal delivery g. Perform basic neonatal resuscitation 2. CEmOC signal functions a. Perform surgery Perform blood transfusion	Personnel	2	3
**15**	Organization and delivery of primary healthcare services in Petropolis, Brazil	Macinko J, Almeida C, Oliveria ES, Sa P K [42]	International Journal of Health Planning and Management	Brazil	Mixed Method [Quantitative (facility survey) Qualitative(validated participant selection)]	Primary care facility and Family care centres	33 care facilities	Attributes of Primary care systems	1. Accessibility 2. First contact 3. Longitudinality 4. Comprehensiveness 5. Coordination 6. Family-focused 7. Community orientation 8. Provider characteristics	Centre	2	3

*Qualitative article with sound theoretical framework derived from literature

### Quality of measures

Fourteen articles had a good description of the measurement properties. The quality of the measures varied. Measures in eleven articles scored 3, two articles scored 2 and two articles scored 1 (Table 1). None of the measures was tested for validity with good results.

### Description of the PHC performance measures

**Fig. 2:**
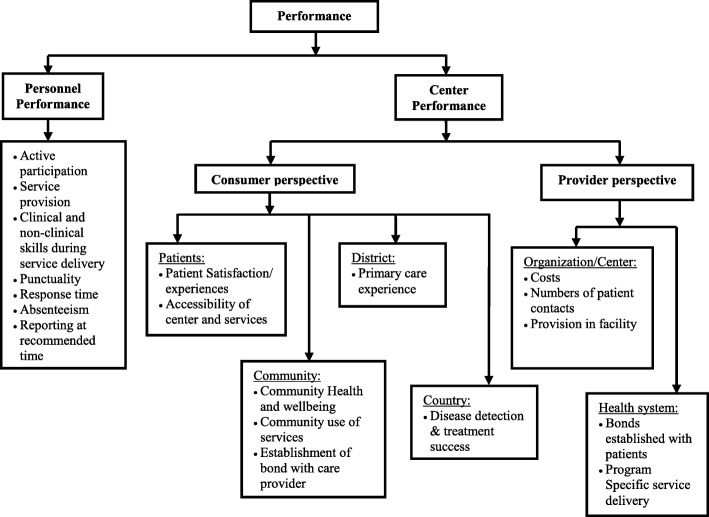
Overview of Primary Healthcare Performance Assessment in Developing Countries

Empirical research on PHC performance measures was published from Brazil, India, Papua New Guinea, Egypt, Bangladesh, Armenia, Malawi, Tanzania, Rwanda, Serbia and other developing countries. A descriptive analysis revealed that PHC performance was distinguished as individual staff/personnel performance and that of the centre/health system performance with a focus on consumer’s and/or provider’s perspectives. Figure 2 depicts the perspective and level of assessment.

### Method of performance assessment

Nine articles included in this review used quantitative methods, one article had exclusively employed qualitative methods and five had employed mixed methods. The methods of data collection included were interviews with clients, health workers, key informants and/or stakeholders [30–38], focus group discussions [30], direct observation [35, 39], facility-based survey [34, 35, 40, 41], and secondary data analysis (records on costs, infrastructure, service provision, health indicators, number of patients served and other patient details) [30, 31, 34, 39, 42].

### Performance of the professionals

Five studies evaluated the personnel performance assessment (nurses, midwives, management team, community health workers and medical professionals). The assessment considered competency/clinical services and non-competency/non-clinical service-based components. The personnel performance was assessed using the Quick Investigation of Quality tool [34] (Fort and Voltero 2004). Adherence to the protocol was the most common method for competency based personnel performance assessment [30, 33, 35, 36, 38]. For the non-competency based personnel performance assessment, the measures used were punctuality, response time, absenteeism, time of reporting to work, time spent with patients, waiting time and cleanliness [33, 36]. Reproductive healthcare delivery was evaluated in all the papers [30, 33, 35, 36, 38].

It was found that autonomy of the team, availability of manpower, clarity in the job description, roles and responsibilities, working conditions, workload and environment, the level of motivation, their education and training along with good supervisory practices contributed to the performance of healthcare personnel [33, 38].

### Performance of the health system/Centre

Of the 15 articles selected, 9 studies assessed the performance of the centre or the health system [31, 32, 34, 36, 37, 39–44]. Satisfaction of patients, community, care providers and other stakeholders were the most common measures utilized for centre/health system performance assessment. The performance of the health system was assessed from the provider’s perspective (4 articles) [31, 34, 37, 39], the consumer’s perspective (4 articles) [32, 40, 42, 43] and both perspectives (2 article) [41, 44] at various levels.

### Perspective and structural levels of performance

The target groups in the studies were individual patients, local community, district or country level. Health care personnel such as nurses, doctors and health managers along with other key stakeholders like local governing bodies were included. From the respondent’s perspective, performance was defined as satisfaction, accessibility of the centre, case detection and success of the treatment provided in the country, care experiences, establishment of patient’s bonds for treatment success and organizational care. Centre performance assessment was assessed as provider and patient bonding leading to therapy compliance and treatment success. This was also assessed in terms of costs and effectiveness of the services provided, the satisfaction of the providers and other stakeholders on the availability of resources and functioning of the centre. The scope of the centre performance assessment was at the structural levels of centre, community, district or country.

### Comparison with the WHO aspects of health systems performance assessment

The measures described in the articles were compared with the six aspects of the WHO health systems performance assessment framework (Table 2) [29]. In the articles included, measures for three aspects (overall level of responsiveness, distribution of responsiveness and distribution of resources) were considered in relation to specific diseases/services, though the WHO framework uses system-specific measures. Measures for disease/service specific WHO aspects (overall level of health, distribution of health in population and distribution of financial contribution) were less well represented in the fifteen articles.

**Table 2:** Measures categorized as per WHO aspects of health systems performance assessment

WHO aspects Measures	Overall Level of Health	Distribution of Health in Population	Overall Level of Responsiveness	Distribution of Responsiveness	Distribution of Resources	Distribution of Financial Contribution
Global standard: Measures from WHO aspects of performance assessment [15]	1. Mortality rate: age and specific 2. Morbidity rate: age and disease specific 3. Maternal Mortality Ratio 4. Infant Mortality rate and Neonatal Mortality rate 5. DALE/DALY/HALE 6. Life expectancy 7. Disease and disability prevalence	1. Immunization coverage 2. Antenatal care 3. Natal care: percentage of deliveries attended by trained personnel 4. Growth monitoring 5. Contraceptive prevalence rate 6. Emergence of communicable disease	1. Quality of care 2. Client satisfaction	1. Availability and use of facilities 2. Human resources for health	1. Human resources 2. Healthcare facilities	1. Government health budget 2. Contribution of NGOs/donors on health services 3. Contribution for insurance 4. Individual out-of-pocket expenditure 5. Measurement of health system efficiency: Per capita expenditure 6. Education/ average years of schooling
Measures from review of articles	• Case detection and Treatment Success Rate [42] • Incidence, prevalence and mortality by disease [42] • Community health [31] • Number of deliveries and maternal mortality figs. [33]	• Coverage of general / program specific services [37, 39, 42] • Community health [31]	• Client experience and satisfaction with quality of care: privacy, doctor client interaction, nurse client interaction, staff attitude, explanation, economic feasibility, availability of drugs, cost of service/drugs [32, 34, 36, 42] • waiting time, time with provider, cleanliness, environment [32, 36] • Facility hours, privacy, patient records, health education [34] • Provider and key informant satisfaction with budget, costs, financial resources, facility and local management characteristics and linkages [34] • Number of patients served [30, 34] • Provider client bonding [42, 44] • Program implementation [39] • Services / skill item performance: quality, quantity, efficiency, problem solving capacity, adaptability [30] • Primary health care experiences with respect to: access, gate keeping/ first contact, comprehensiveness, coordination, family focus [40, 41] • Community orientation, Provider characteristics [36, 40] • Attributes of primary care- Longitudinality, comprehensiveness, coordination [40, 41]	• Use of antenatal, childbirth, immunization services, environmentally induced disease like malaria, pneumonia, diarrhoea [31, 32, 39] • Utilization of maternal and child health services like abortion, under 5 year mortality, preventive & curative services, antenatal care, delivery services, family planning services [32] • Availability and coverage of care [37, 40] • Attributes of primary care- first contact, community orientation, provider characteristics [41]	Human resources: • Skill sets: prenatal, intra natal and post-natal care protocol [35, 38] • Active participation in core activities [30, 38] Attitude and characteristics of provider- punctuality, response time, absenteeism, supervisory practices, collaboration, co-ordination, community orientation, compliance to health needs, health activities performed [33, 36, 37, 39, 40, 41]and collaboration Facility provision: Availability of supplies, maintained registers, cleanliness [33, 34, 36, 37] Accessibility [34, 40, 41, 43] • Organization accessibility (building size to patient load) • Economic accessibility • Geographic accessibility Basic Infrastructure: building structure, toilet, clean running water, electricity, communication, equipment and instrument, furniture, drugs and supplies [34]	• Funding received /expenditure [34, 42] • Cost effectiveness [34]

## Discussion

The aim of this review was to describe the measures of PHC performance assessment used in developing countries as published in the empirical literature and to compare them with the WHO framework for health systems performance assessment. The fifteen articles that were considered provided scarce information on measurement quality and covered limited aspects of PHC performance when compared to the WHO health systems performance assessment framework. Measures were found both at the level of the performance of professionals and of the centre, and further measures that addressed the satisfaction of the performance by stakeholders were found. These measures will be discussed below.

### Performance of professionals

Personnel performance was based on observation methods, assessed competency and non-competency based tasks. This correlated highly with patient satisfaction, an indicator of centre performance, as well as that of availability of resources, support and culture of the organization [45]. The investigators used the ‘Quick Investigation of Quality’ tool that had been validated earlier [45, 46]. Such validated tools for observation of professional performance provide a quick and effective method for assessment of PHC personnel performance in a developing country. As reproductive and child health is one of the main focus areas of primary healthcare and an important health indicator, it is hence appropriate to use this in the evaluation of PHC performance as well.

### Performance of the Centre

The performance of the centre was assessed using the Primary Care Assessment Tool [40, 41, 43]. This tool was validated in a developing country for family healthcare [41, 47]. It is interesting to note that this instrument could be used to assess the performance from the consumer’s as well as from the provider’s perspective. This tool provides an extensive list of surveys on different attributes of PHC. Based on the requirement of the evaluation, one could consider specific components. In other studies, measures such as costs, patients served and effectiveness along with the client satisfaction were identified. Cost effectiveness is an appropriate measure that can be used across all PHCs [48] and thus, helps in making right choices by identifying the most effective service or intervention or centre [48].

### Satisfaction with performance by various stakeholders

Satisfaction of various stakeholders including patients and providers is an accepted standard in performance assessment as they are interrelated. Community participation in the functioning of the PHC highly influences the performance of PHC and its personnel, so it could be included as a component in the PHC performance assessment [34].

### WHO aspects of performance assessment

The current practices of PHC performance assessment in developing countries were analysed with the WHO framework for performance assessment [17, 29]. The WHO framework is completely focused on output and outcome, with the structure and process considered intrinsic to the system. However, for ease and clarity, many authors have used the Donabedian model for assessing performance in primary health care [19, 23, 26, 49]. The current review highlights the limited representation of the performance measures in relation to aspects of the WHO framework (Table 2). In the articles, even the tools used to assess personnel performance (Quick Investigation of Quality tool) and centre performance (Primary Care Assessment Tool) did not represent measures from three aspects: overall level of health, distribution of health in the population and the distribution of the financial contribution [40, 41, 47].

Due to lack of resources and data [10, 18], covering the complete list of WHO aspects to the full extent will be difficult for developing countries. However, a concise list of measures with an appropriate representation of six WHO aspects and requiring minimum resources and data, needs to be developed for PHC performance assessment in developing countries. If the assessment uses standardised measures useful comparisons across regions/countries would be possible. Since the search results indicated that a very small number of articles were published, further research needs to be conducted in the developing countries on PHC performance assessment, enabling cross learning and knowledge base enhancement [21].

### Discussion on methodological quality of articles

Although the number of articles in this review was limited, the studies covered diverse countries and continents. Assessing the quality of the papers was a challenge as articles used qualitative, quantitative and mixed methodology. The articles demonstrated reliability and validity methods such as correlating provider and user experiences, data/method triangulation, standardised tools/training of researchers. Hence, there is a need for research in developing countries to establish quality of measurement and standardisation of PHC performance assessment using rigorous statistical methods.

### Implications for future research

PHC performance assessment in developing countries is an emerging field but it is fragmented at present. Though human resource for health is a component of the WHO framework, the various sub-components to be included are not clear. Evaluation of the PHCs is an on-going exercise, but yet there is no established standard for assessing performance. Studies have shown that the performance of PHC depends on several things, one critical factor being personnel performance [21, 29, 50–52].

### Implications for practice

What is measured, can be controlled. However, not all aspects of PHC performance were yet covered. Measures such as skill sets for execution of healthcare focusing on mother and child health, are already well available. They were emphasised in five studies of our review and this is in line with Millennium Development Goals and Sustainable Development Goals [1, 2]. However, other aspects like coverage/distribution of health, overall health of the population, distribution of financial contribution, including program specific achievements, various service components and process of primary care delivery are still not covered. The performance measures should be appropriate and adequate enough to enable accurate assessment on an ongoing basis to aid in monitoring and efficient management of the personnel/system. There is urgency to develop new and additional measures for PHC performance in developing countries. PHC performance is a matter of immense importance for policy agenda and political priorities [53].

## Conclusions

Although developing countries may have difficulties in applying the entire WHO framework, the current measures for assessment of PHC performance published in scientific journals are limited in scope and lack validation. The standard health indicators for the overall level of health and for the distribution of health in the population were represented least. A comprehensive assessment of primary healthcare can be achieved by integrating personnel performance with that of centre performance. Representation/inputs from both sides of the service delivery, the management and the consumer/public, that is, including the provider’s perspective and consumer’s perspective are vital. From this review, it can be concluded that existing measures for PHC performance assessment in developing countries need to be validated and concise measures for neglected aspects need to be developed.

## Additional file

Additional file 1: **Appendix 1.** Search Strategies. **Appendix 2.** Additional Search Strategies. (DOCX 105 kb)

## Abbreviations

PHC: Primary Health Centre
WHO: World Health Organization
