# Supplementary material for: Primary health Centres’ performance assessment measures in developing countries: review of the empirical literature
Source: BMC Health Serv Res. 2018 Aug 9;18:627. doi: 10.1186/s12913-018-3423-0 (PMC6085632; doi:10.1186/s12913-018-3423-0)
Supplement: Supplementary file 1 — Appendix 1. Search Strategies. Appendix 2. Additional Search Strategies. (DOCX 105 kb) [file 12913_2018_3423_MOESM1_ESM.docx]

**Appendix 1: Search Strategies**

**A1.1 JSTOR**

**Date Search: 27/2/2015**

|  | **Key concept 1**  **Primary Health Centre**  And  OR↓ | **Key concept 2**  **Performance Assessment**  And | **Key concept 3**  **Developing country** |
| --- | --- | --- | --- |
|  | Health Centre | OR↓ | OR↓ |
| **Synonyms/different spelling/Alternative/ Controlled keyword** | In Abstract | In Abstract | In Abstract |

**Search limits:**

| Study Type | Articles and Reviews |
| --- | --- |
| Age range | Date range: 1979/01/01 to 2014/12/31 |
| Language | English |
| Disciplines | Narrowed by Discipline and/or publication title: Business and Economics, Health Policy, Health Sciences, Management& Organizational Behaviour, Psychology, Public Health , Social Science |
| Source/Database | JSTOR |

**Search Results: 617**

**A1.2 EMERALD INSIGHT**

**Date Search: 27/2/2015**

|  | **Key concept 1**  **Primary Health Centre**  OR↓  And | **Key concept 2**  **Performance Assessment**  OR↓  And | **Key concept 3**  **Developing countries**  OR↓ |
| --- | --- | --- | --- |
|  | Health Centre | performance |  |
| **Synonyms/different spelling/Alternative/ Controlled keyword** | In Abstract | In Abstract | In Abstract |

**Search limits:**

| Content Type | All content, early cites, Backfiles |
| --- | --- |
| Years Searched | January 1979 to December 2014 |
| Source/Database | Emerald Insight |

**Search Results: 1133**

**A1.3 PSYCINFO**

**Date Search: 27/2/2015**

|  | **Key concept 1**  **Primary Health Centre**  OR↓ | **Key concept 2**  **Performance Assessment**  And  OR↓  And | **Key concept 3**  **Developing country** |
| --- | --- | --- | --- |
|  | Health Centre | Performance | OR↓ |
| **Synonyms/different spelling/Alternative/ Controlled keyword** | In Abstract | In Abstract | In Abstract |

**Search limits:**

| Search type | Boolean/Phrase |
| --- | --- |
| Study Type | Peer reviewed |
| Years searched | : 1979-2014 |
| Publication date: | 19790101-20141231 |
| Publication type | Peer Reviewed Journal |
| Language | English |
| Population group | Human |
| Source database | PSycInfo |

**Search Results: 642**

**A1.4 PUBMED**

**Date Search: 27/2/2015**

|  | **Key concept 1**  **Primary Health Centre**  OR↓  And | **Key concept 2**  **Performance assessment**  OR↓  And | **Key concept 3**  OR↓ |
| --- | --- | --- | --- |
|  |  |  |  |
| **Synonyms/different spelling/Alternative/ Controlled keyword** | In Abstract | In Abstract | In Abstract |

**Search limits:**

| Years searched | 1979/01/01-2014/12/31 |
| --- | --- |
| Language | English |

**Search Results: 373**

**A1.5 EMBASE**

**Date Search: 27/2/2015**

|  | **Key concept 1**  **Performance assessment**  OR↓  And | **Key concept 2**  **Health**  OR↓  And | **Key concept 3**  OR↓ |
| --- | --- | --- | --- |
|  |  |  |  |
| **Synonyms/different spelling/Alternative/ Controlled keyword** | In Abstract | In Abstract | In Abstract |

**Search limits:**

| Study Type | Journal article |
| --- | --- |
| Years searched | 1979-2014 |
| Publication type | Journal |
| Language | English |
| Subject | Human |
| Sources/Database | Abstract records from Embase |

**Search Results: 262**

**Appendix 2: Additional Search Strategies**

**A2.1 JSTOR**

**Date Search: 15/10/2016**

|  | **Key concept 1**  **Primary Health Centre**  OR↓  And | **Key concept 2**  **Performance Assessment**  OR↓ | **Key concept 3**  And  **Developing country**  OR↓ |
| --- | --- | --- | --- |
|  | Health Centre |  |  |
| **Synonyms/different spelling/Alternative/ Controlled keyword** | In Abstract | In Abstract | In Abstract |

**Search limits:**

| Study Type | Articles and Reviews |
| --- | --- |
| Age range | Date range: 2015/1/1to 2016/10/15 |
| Language | English |
| Disciplines | Narrowed by Discipline and/or publication title: Business, Economics, Health Policy, Health Sciences, Management & Organizational Behaviour, Psychology, Public Health , Sociology |
| Source/Database | JSTOR |

**Search Results: 430**

(((ab:(Primary Health Centre) OR ab:(Health Centre)) AND ab:(Performance Assessment)) AND ab:(Developing country)) AND la:(eng OR en) AND disc:(health-discipline OR healthsciences-discipline OR sociology-discipline OR economics-discipline OR manorgbeha-discipline OR business-discipline OR publichealth-discipline OR psychology-discipline)

**A2.2 EMERALD INSIGHT**

**Date Search: 15/10/2016**

|  | **Key concept 1**  And  **Primary Health Centre**  OR↓ | **Key concept 2**  **Performance Assessment** | **Key concept 3**  And  **Developing countries**  OR↓ |
| --- | --- | --- | --- |
|  | Health Centre | performance  OR↓ |  |
| **Synonyms/different spelling/Alternative/ Controlled keyword** | In Abstract | In Abstract | In Abstract |

**Search limits:**

| Include | All content, Accepted articles, Back files |
| --- | --- |
| Narrow by Content Type | Articles and Chapter |
| Years Searched | January 2015 to October 2016 |
| Source/Database | Emerald Insight |

**Search Results: 540**

**A2.3 PSYCINFO**

**Date Search: 15/10/2016**

|  | **Key concept 1**  **Primary Health Centre**  OR↓ | **Key concept 2**  And  **Performance Assessment** | **Key concept 3**  And  **Developing country** |
| --- | --- | --- | --- |
|  | Health Centre | Performance  OR↓ | OR↓ |
| **Synonyms/different spelling/Alternative/ Controlled keyword** | In Abstract | In Abstract | In Abstract |

**Search limits:**

| Search type | Boolean/Phrase |
| --- | --- |
| Study Type | Peer reviewed |
| Years searched | : 1979-2014 |
| Publication date: | January 2015 to October 2016 |
| Publication type | Peer Reviewed Journal |
| Language | English |
| Population group | Human |
| Source database | PSycInfo |

**Search Results: 94**

**A2.4 PUBMED**

**Date Search:15/10/2016**

|  | **Key concept 1**  **Primary Health Centre**  OR↓ | **Key concept 2**  And  And  **Performance assessment**  OR↓ | **Key concept 3**  OR↓ |
| --- | --- | --- | --- |
|  |  |  |  |
| **Synonyms/different spelling/Alternative/ Controlled keyword** | All field | All field | In Abstract |

**Search limits:**

| Years searched | 2015/01/01 to 2016/10/15 |
| --- | --- |
| Language | English |

**Search Results: 242**

(Primary[All Fields] AND Health[All Fields] AND Centre[All Fields]) AND (Performance[All Fields] AND assessment[All Fields]) AND ("2015/01/01"[PDAT] : "2016/10/15"[PDAT])

**A2.5 EMBASE**

**Date Search: 15/10/2016**

|  | **Key concept 1**  And  **Performance assessment**  OR↓ | **Key concept 2**  And  **Health**  OR↓ | **Key concept 3**  OR↓ |
| --- | --- | --- | --- |
|  |  |  |  |
| **Synonyms/different spelling/Alternative/ Controlled keyword** | In Abstract | In Abstract | In Abstract |

**Search limits:**

| Study Type | Journal article |
| --- | --- |
| Years searched | 2015-2016 |
| Publication type | Journal |
| Subject | Human |
| Sources/Database | Embase |

**Search Results: 27**
